# The Surveillance After Extremity Tumor Surgery (SAFETY) Pilot International Multi-Center Randomized Controlled Trial

**DOI:** 10.3390/curroncol32120686

**Published:** 2025-12-04

**Authors:** Hadia Farrukh, Patricia Schneider, Tess Hudson, Victoria Giglio, Ricardo Gehrke Becker, Samir Sabharwal, Kimmen Quan, Valerie Francescutti, Mira Goldberg, Sheila Sprague, Michelle Ghert

**Affiliations:** 1 Department of Surgery, McMaster University, Hamilton, ON L8S 4K1, Canadavictoria.giglio@hse.ie (V.G.); francesv@hhsc.ca (V.F.);; 2 Hospital de Clínicas de Porto Alegre, Porto Alegre 90035-903, Brazil; 3 Department of Orthopaedic Surgery, University of Cincinnati, Cincinnati, OH 45267, USA; sabharsr@ucmail.uc.edu; 4 Department of Oncology, McMaster University, Hamilton, ON L8S 4K1, Canada; 5 Department of Orthopaedics, University of Maryland, Baltimore, MD 21201, USA

**Keywords:** soft-tissue sarcoma, surveillance, lung metastasis, recurrence, patient-important outcomes, randomized controlled trial

## Abstract

Soft-tissue sarcomas (STSs) are rare malignancies predominantly found in the extremities. Surgery and radiation are standard treatments, but post-operative pulmonary surveillance, involving clinical visits and thoracic imaging, is crucial due to a high recurrence rate, most commonly to the lungs. Current pulmonary surveillance guidelines lack robust evidence. The Surveillance AFter Extremity Tumor SurgerY (SAFETY) randomized controlled trial is designed to determine the impact of pulmonary surveillance frequency (every three versus six months) and chest imaging modality (CXR versus CT) on patient-important outcomes. The pilot phase assessed feasibility of patient enrolment, protocol adherence, and data quality, as well as aggregate outcomes at two years of follow-up. 100 patients were enrolled from 300 screened patients across 17 international sites. Minor protocol deviations were common. Follow-up, data completeness and data quality met the progression criteria of 85%. Of the 100 patients, 15 died, 21 had metastases, seven had local recurrence and 30 experienced at least one serious adverse event. This SAFETY trial study established feasibility of enrolment and data quality, and confirmed the need to emphasize protocol adherence in sarcoma care. The results of this trial are expected to provide crucial evidence to standardize STS pulmonary surveillance practices, ultimately improving patient management and expectations.

## Simple Summary

Soft-tissue sarcomas are rare cancers that often recur after surgery, most commonly in the lungs. Healthcare professionals routinely follow patients with regular clinic visits and chest imaging, but there is little solid evidence to guide how often to schedule these visits or whether chest x-ray or computed tomography is best. This international pilot study tested two pulmonary surveillance visit schedules (every three versus every six months) and two imaging approaches (chest X-ray versus computed tomography) in people treated for high-grade sarcoma of the upper or lower extremity. The study showed that enrolling patients across many hospitals and collecting high-quality data are feasible, while also revealing common real-world challenges like extra visits for wound issues. These results pave the way for the full trial, which could inform future guidelines, reduce unnecessary testing and anxiety, and help health systems use resources more wisely.

## 1. Introduction

Soft-tissue sarcomas (STSs) are a rare and heterogeneous group of tumors that account for less than one percent of all malignancies, and most commonly occur in the extremities [1]. Surgery and radiation therapy are the standard modalities used in the attempt to cure localized STS, since systemic therapies, though available, are controversial in their effectiveness. Between 40 and 50% of patients will recur after treatment. Metastasis to the lung is the most frequent single location of disease recurrence, occurring in nearly all STS patients that develop metastases [2,3]. Therefore, local and systemic surveillance is initiated with the goal of intervening for potentially curable local recurrences, or to palliatively manage systemic recurrences and, in some cases, prolong survival.

Surveillance to monitor for lung metastases entails visits to outpatient clinics for at least five years after surgery. These visits typically include a clinical history, physical examination, and thoracic imaging with either a chest radiograph (CXR) or a computed tomography (CT) scan, the latter being a more sensitive modality yet incurring a higher cost to the healthcare system and/or the patient. More frequent and sensitive lung imaging will identify pulmonary recurrence earlier and conversely may provide reassurance to patients if imaging is clear. However, frequent and intensive investigations can induce anxiety and increase the risk for false positive results. The incidence of false positives and/or incidental findings is high in sarcoma surveillance [4]. These findings lead to patient stress, unnecessary interventions, and the risk of complications from these interventions. Furthermore, when asymptomatic recurrence is detected, treatment is often intentionally delayed until symptoms occur to maximize patient quality of life.

Although pulmonary surveillance is widely considered an essential aspect of sarcoma management, surveillance strategies have not been well researched, and current guidelines are not evidence-based [5,6]. These guidelines are also quite broad in that follow-up is recommended every three to six months in the first two years with no specific thoracic imaging modality indicated. Several published surveys in the field highlight the uncertainty, variation and clinical equipoise among orthopedic oncologists, with the number of clinic visits ranging from two to 12, the number of CXRs obtained ranging from zero to 13, and the number of chest CT scans ranging from one to eight in the first two years of surveillance [7,8,9]. Given the lack of evidence to guide surveillance, the evaluation of post-operative surveillance strategies has emerged as the highest-ranking research priority in the orthopedic oncology field [10].

The Surveillance AFter Extremity Tumor Surgery (SAFETY) randomized controlled trial (RCT) was designed to determine the effect of pulmonary surveillance strategy on overall survival and other patient-important outcomes following extremity STS surgery. The trial aims to determine the impact of pulmonary surveillance frequency (every three versus six months) and surveillance imaging modality (CXR versus chest CT scan) on outcomes at up to five years of follow-up. The objective of this SAFETY trial study was to determine the feasibility of the definitive SAFETY RCT with the progression criteria of: (1) a rate of 100 patients enrolled per year, and (2) protocol adherence, follow-up and data quality of at least 85%.

## 2. Materials and Methods

### 2.1. Study Design and Setting

The SAFETY trial is a multi-center 2 × 2 factorial randomized controlled trial of patients with an extremity STS who present without metastases and who require surgical management and post-operative pulmonary surveillance for disease recurrence. The SAFETY trial is centrally coordinated by the Methods Centre in McMaster University’s Department of Surgery in Hamilton, Ontario. A total of 17 participating clinical sites enrolled patients. These sites are in Canada, the United States, Austria, Brazil, Italy, and the Netherlands. The study is registered on ClincialTrials.gov, identification number NCT03944798 (https://clinicaltrials.gov/study/NCT03944798 Registered 7 May 2019, accessed on 29 September 2025). The study was approved by the Hamilton Integrated Research Ethics Board (HiREB #16445) and all corresponding ethics/regulatory boards at participating clinical sites.

Each clinical site is appointed a locally responsible investigator to supervise the trial’s local conduct. This investigator assesses the eligibility of patients, formulates a site-specific enrolment plan, and completes a screening form for all patients aged 18 and above who undergo surgery for their high-grade STS, regardless of their study eligibility. Eligible patients undergo screening and consent during their clinic visit after completing all treatment and having their surgical wound deemed healed, at which point discussion of the post-treatment surveillance plan is initiated. The process of obtaining and recording informed consent adheres to local Good Clinical Practice guidelines and complies with the relevant ethics and/or regulatory committee and, where applicable, the Health Insurance Portability and Accountability Act.

The Methods Center ensures that the Site Principal Investigator and Research Coordinator have received appropriate study-specific training. The Methods Center also remotely conducts the following ongoing monitoring activities: (1) Review quality control reports from the iDataFax electronic data capture system to identify sites with unacceptable amounts of missing data or unresolved queries; (2) Review enrolment reports to identify low-enrolment sites and those which had not been submitting screening data; (3) Review the tracking database to identify any inconsistencies between the randomization system and the submitted Case Report Forms (CRFs) with respect to surveillance allocation.

### 2.2. Participants

All patients 18 years of age or older who present for surgery of a high-grade STS are screened for participation. Eligible patients must have the following: (1) a primary extremity grade II or III STS, that is (2) equal to or greater than five centimeters in size (American Joint Committee on Cancer Stage IIIA or IIIB), (3) have undergone surgical excision of the tumor with curative intent and with no evidence of gross residual disease (tumors with R1 margins also eligible), and (4) have completed any adjuvant therapy. Patients with indeterminate pulmonary nodules on chest CT scan at presentation undergo repeat CT imaging once local treatment is completed. If the repeat imaging rules out metastases (due to nodule spontaneous regression or stability over time), the patients will be deemed eligible for the trial. Patients who have been diagnosed with any of the following are not eligible: myxoid/round cell liposarcoma; extra-skeletal Ewing’s sarcoma; a genetic syndrome with an elevated risk of malignancy; or a co-morbid condition that has a life expectancy of less than one year. Further details of the inclusion and exclusion criteria have been previously published [11].

### 2.3. Randomization

Pulmonary surveillance allocation is determined using a 2 × 2 factorial design. While the unit of randomization is the patient, each study patient is randomized twice: once to determine their specific surveillance frequency allocation (clinic visit every three or six months) and once to determine their specific imaging modality allocation (chest CT scan or CXR). A centralized web-based randomization system (www.randomize.net) ensures the concealed randomization of patients. Treatment allocation is determined using random variable block sizes to avoid substantial imbalance in the number of patients assigned to each group. To ensure balance across groups for any key prognostic and treatment-related variables, such as regional differences in STS management, randomization is stratified by clinical site and peri-operative chemotherapy.

### 2.4. Interventions

Patients are randomized to one of four groups:

(1) clinic visit and CXR every three months for two years;
(2) clinic visit and CXR every six months for two years;
(3) clinic visit and chest CT scan every three months for two years; or
(4) clinic visit and chest CT scan every six months for two years.

Clinic visits include a detailed clinical history including information on any events such as recurrence and other serious adverse events as well as their treatment, physical examination, and thoracic imaging, the latter of which is conducted as per the group to which they were randomized (CXR or chest CT).

### 2.5. Outcomes and Assessment

Pilot Phase: The pilot phase progression criteria identified were: (1) participant enrolment in the trial, (2) participants and clinical sites’ adherence to the study protocol, and (3) participant follow up and data quality (Table 1). Overall, the trial would be considered feasible with a rate of participant enrolment of approximately 100 patients per year with the full complement of clinical sites open (9 patients per month), and protocol adherence, follow-up, and data quality of at least 85% [11].

At the time of trial initiation, the primary outcome for the definitive SAFETY trial was overall survival as defined by death from any cause at five years post randomization. The primary outcome was subsequently revised as described later in this manuscript. The secondary outcomes include (1) serious adverse events (SAEs)—defined as adverse events that result in congenital anomaly/birth defects, hospitalization, significant disability, or death, or are categorized as life-threatening or an important medical event by the investigator—(2) patient reported anxiety, satisfaction and quality of life using the Patient-Reported Outcomes Measurement Information System (PROMIS^®^) Cancer-Anxiety, PROMIS^®^ Satisfaction with Social Roles, and EuroQol Group-5 Dimensions (EQ-5D)™ questionnaires that have been validated in the oncologic population [12,13], as well as (3) recurrence-free and (4) metastasis-free survival. These patient-important outcomes were not analyzed and are not reported in this manuscript, as we designed the study a priori to not report interim results due to the risk of overestimating treatment effect.

Patient follow-up and assessment for the two-year surveillance protocol are shown below in Table 2.

Data and Safety Monitoring Board: The SAFETY Trial Data and Safety Monitoring Board (DSMB) comprises independent individuals appointed according to standard guidelines. The members of the DSMB collectively judge the strength of the evidence and the absolute magnitude and seriousness of any safety signals. The SAFETY DSMB includes experts in orthopedic oncology, biostatistics, and an independent patient representative. The DSMB receives quarterly reports on SAEs and meets annually to review the SAFETY trial data.

### 2.6. Statistical Analysis and Study Sample Size

We report the total number of participants enrolled per month, as well as patient baseline characteristics. Each participating site keeps a Screening Log of included, excluded, and missed patients. We report the number of participants who miss visits, miss thoracic imaging, have unscheduled thoracic imaging or thoracic imaging different than directed by the protocol, and those who withdraw or are lost to follow-up. Aggregate clinical outcomes (mortality, local recurrence, metastasis, and SAEs) are reported. All data is reported using descriptive statistics—reported as counts (percent) for categorical variables and mean (Standard Deviation—SD) for continuous variables. There were no continuous non-normally distributed variables in the dataset. We report the proportion of completed CRFs as descriptive data. All analyses were conducted using Excel by the first two authors.

We considered the SAFETY pilot phase to be complete after the first 100 enrolled patients completed the two-year intervention phase surveillance. The original pilot sample size was initially calculated using the expected rate of protocol adherence (85%) at 195 [11]. However, given subsequent revisions to the protocol, the SAFETY trial Steering Committee decided in November 2023 to publish this study after the first 100 patients completed their allocated two-year surveillance phase, as this was sufficiently robust to assess the progression criteria.

## 3. Results

### 3.1. Enrolment

**Figure 1:**
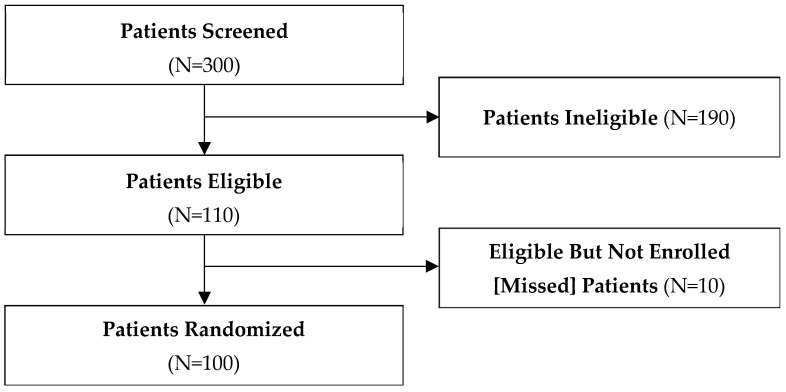
Patient flow diagram.

We screened 300 patients across 20 clinical sites in Canada, the United States, Argentina, Austria, Brazil, Italy, and the Netherlands between November 2019 and December 2021 (Table 3). Of these, 100 were eligible and included in this study from 17 clinical sites, 10 were eligible and missed, and 190 were excluded (Figure 1). Of those excluded, 42 were otherwise eligible but declined to participate, and the remainder did not meet all eligibility criteria. The most common reason provided by patients for declining to participate in the study was concern for randomization to less intense surveillance.

**Figure 2:**
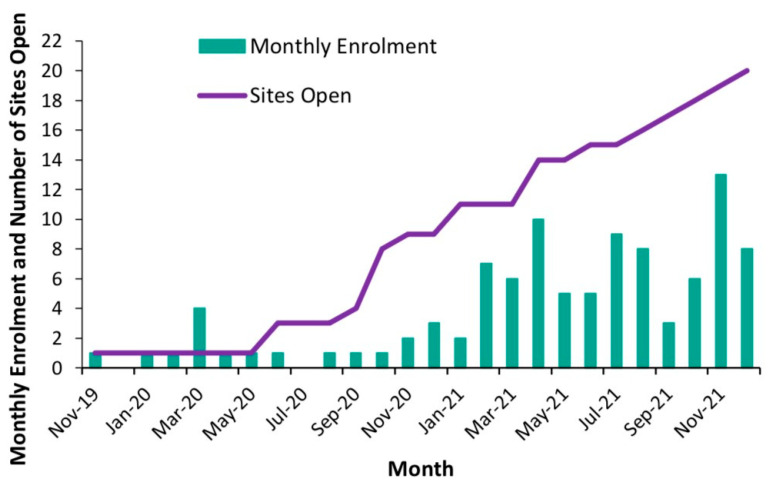
Monthly enrolment and number of sites open during pilot study.

The number of participants enrolled at each clinical site ranged between zero (three sites that became enrolment ready later) and 37 (one site). The average monthly enrolment was 1.3 (SD 1.1) in the first year of enrolment (2020) and 6.8 (SD 3.0) in the second year of enrolment (2021), with nine clinical sites open for enrolment by the end of the first year and 20 open for enrolment by December 2021 (Figure 2). With 14 further clinical sites imminently completing the startup phase, the enrolment of 9 patients per month (at least 100 per year) was deemed feasible with the expected 34 sites open for enrolment.

### 3.2. Demographics

The baseline characteristics of the 100 patients are presented in Table 4. Briefly, there were 52 who self-identified as men and 48 who self-identified as women, and their mean age at the time of enrolment was 59.8 years (SD: 15). The most common histologic diagnoses were undifferentiated pleiomorphic sarcoma (26%) and myxofibrosarcoma (20%), and the most common tumor location was the thigh (33%). In total, 6% underwent neoadjuvant chemotherapy and 77% underwent neoadjuvant radiation therapy, while 3% underwent adjuvant chemotherapy and 9% underwent adjuvant radiation therapy.

### 3.3. Aggregate Clinical Outcomes

Of the 100 patients included in this study, a total of 24 (24%) patients suffered a local and/or systemic recurrence—three patients experienced a local recurrence only, 17 patients experienced systemic recurrence only, and four experienced both local and systemic recurrences. Of the seven patients who experienced a local recurrence, four were detected by standard MRI surveillance imaging, one by CT imaging, one by physical exam, and one was first identified by the patient. Fifteen patients (15%) died—14 (14%) within the first two-years of surveillance and one (1%) within the following six months. The most common cause of death was systemic recurrence.

Thirty-nine instances of systemic recurrence were reported in the 21 patients with systemic recurrence—20 instances of metastases to the lung, four instances of lymph node metastases, four instances of bone metastases, three instances of liver metastases, and eight instances of metastases to other anatomical locations. Metastases to locations other than the lungs were usually worsening of disease first located in the lungs, and were either detected through CT chest, abdomen, and pelvis (standard surveillance imaging for recurrent sarcoma), incidental findings, or further investigation of patient-reported symptoms. Eighteen systemic recurrences were detected within the first six months of surveillance, four between six months and one year, nine between one year and 18 months, seven between 18 months and two years, and one between two years and 30 months. Finally, 30 patients (30%) experienced a total of 52 SAEs (see Table S1 for listing). The most common SAE was a surgical wound complication.

The SAFETY trial’s DSMB met annually in 2022, 2023, and 2024, and agreed that the reported events were within the realm of what was to be expected in this patient population. As such, they unanimously approved the continuation of the trial as designed.

### 3.4. Protocol Adherence

A total of 21 patients deviated from the protocol after they were diagnosed with a systemic or local recurrence, which required the use of a thoracic imaging modality outside of the allocated protocol, additional clinic visits with the orthopedic oncologist, and/or additional unscheduled thoracic imaging. These patients were deemed ‘off-protocol’ for the remainder of their follow-up period in the trial or until surveillance was restarted. Only two technically ineligible patients were enrolled in the trial—both had tumors slightly less than five centimeters in size (Table 5), and while their eligibility will be adjudicated by the Central Adjudication Committee prior to final data analysis, site personnel are continuing to follow them as per the intention-to-treat principle.

For the 100 patients in this study, the protocol at randomization dictated an aggregate total of 600 clinic visits and 600 thoracic imaging studies over two years. Given this large number of potential protocol deviations, at least one clinic visit and/or thoracic imaging study protocol deviation was reported for 55 patients who were scheduled to be ‘on protocol’. The most common protocol deviation among participants was at least one unscheduled clinic visit with the orthopedic oncologist that was in addition to the regularly scheduled surveillance visit—this was reported for 33/55 (60%) patients, 13 of whom had wound-healing problems post-surgery. Twenty (36%) patients missed a scheduled thoracic imaging study, and 15 (27%) missed a scheduled surveillance visit with the orthopedic oncologist. Eleven (20%) patients had unscheduled thoracic imaging in addition to their regularly scheduled chest imaging, and only three (5%) patients received chest imaging using a modality other than that to which they were randomized.

### 3.5. Follow-Up and Data Quality

Of the 100 patients enrolled in this study, 15 died, six withdrew consent (two after their two-year follow-up period), and one was withdrawn due to frustration with the healthcare system, but did not express the wish to withdraw from the study. There were no patients considered lost to follow-up, as the research staff are advised to continue attempts to contact the patient for the duration of the five-year follow-up period.

As of June 2024, 78 of the 100 patients are in active follow-up (have not died or withdrawn). Of these patients, 70 (70%) have completed two years of follow-up, 23 (23%) have completed three years of follow-up, and seven (7%) patients have completed four years of follow-up (Table 6). No patients have reached the 54- and 60-month follow-up time points. Of the patients that were eligible for follow-up at each time point, complete data for the two-year intervention phase was collected for 94% at six months, 96% at 12 months, 85% at 18 months, and 85% at 24 months. Questionnaires for Patient-Reported Outcome Measures (PROMs) were missed for 9% of patients at six months, 6% at 12 months, 7% at 18 months, and 11% at 24 months. Data on completeness of follow-up is not reported for the three-month time points to adhere to the principle of blinding to treatment allocation (clinical visits at three- vs. six-month intervals).

## 4. Discussion

### 4.1. Summary of Findings

This SAFETY trial study enrolled 100 patients within the expected timeframe in the context of staggered clinical site start-up. Data completeness met the progression criteria. Of the 100 patients, 21 were taken off their surveillance protocol by their treating physician due to the diagnosis of metastatic disease and/or local recurrence. Fifty-five patients followed their surveillance protocol imperfectly, most commonly due to unscheduled visits to their orthopedic oncologist for post-operative wound complications. However, the vast majority of the imaging studies ordered, and visits scheduled for the 100 patients over the two years of surveillance were considered protocol adherent. There was no loss to follow up. The overall death rate was 15%, while the rates of metastatic disease, local recurrence, and SAEs were 21%, 7%, and 30%, respectively.

### 4.2. Feasibility Implications for the Definitive SAFETY Trial

One feasibility consideration prior to embarking on the SAFETY trial was potential objections raised by ethics boards to the less intense surveillance allocations. However, 20 clinical sites successfully opened to enrolment in the pilot phase, and a further 14 clinical sites have opened in the definitive phase of the study. No clinical sites encountered insurmountable obstacles related to ethics approval. This is likely because all surveillance arms fall within standard surveillance guidelines [5,6], although more intense surveillance is a common approach [7,8,9]. In addition, there remains no evidence that more intense surveillance improves patient outcomes in solid cancers [14,15,16]. The current research question was considered a top priority in the field [10], and therefore widespread passion for the study and clinical site investigator dedication may have assisted with successful ethics approval submissions. The SAFETY trial DSMB has continually recommended that the trial continue as designed, indicating no concerning safety signals in the study patient population.

The acceptance of STS patients to pulmonary surveillance regimens allocated by randomization was previously explored through survey work by our group and found to be as high as 85% [17]. However, real-life scenarios involving the diagnosis of a high-risk disease may not result in the same outcomes as survey research, and individual patient preference will likely play a role in the choice of surveillance intensity [18]. In fact, of the 142 patients screened who were otherwise eligible for participation, 42 did not agree to participate, with the most common reason being that they were concerned that they would not receive sufficient surveillance. Nevertheless, over 2/3 did agree to participate. These numbers have informed the definitive phase of the study, in that a further 14 clinical sites were invited to participate in the trial to increase the average monthly enrolment from 6.8 per month to closer to the expected 9 patients per month.

Finally, we found that protocol deviations were common. Additional clinic visits were most often due to the need for a surgical wound evaluation, although other deviations, such as missed clinic visits and missed lung imaging, were also encountered. Additional or improper imaging was most often completed to manage equivocal changes or to assess respiratory illnesses. These protocol deviations are not unexpected, given the complexity and length of the study protocol involving two full years of adherence to scheduled patient interactions with the healthcare system. Scheduling conflicts due to the COVID-19 pandemic, patient preferences for time off for vacation, and other complications in real-life scenarios often lead to imperfect adherence to clinical follow-up [19]. Finally, sarcoma surgery is high-risk and extra clinic visits for wound assessments are expected. The DSMB felt that these minor protocol deviations were acceptable and do not specifically affect the assessment of pulmonary surveillance. Despite this, we were able to maintain data quality of 85% or higher in the first two years of follow-up, as we had aimed [11]. These findings have informed the definitive phase of the SAFETY trial, as all sites are reminded with regular data queries to maximize patient adherence to the protocol. Ultimately, the pragmatic nature of the SAFETY trial aligns with the day-to-day functioning of healthcare systems and the individual personal lives of the patient participants.

Although reasonable, there are implications of protocol deviations on potential systemic bias. In a randomized trial of cancer surveillance requiring frequent follow-up visits, there is surveillance bias, which may increase incidental findings (i.e., SAEs reported in the trial) and may inflate perceived survival benefits for one treatment arm [20]. Additionally, patients in the more intensive surveillance treatment arm that requires frequent visits and imaging may be more likely to withdraw consent due to commitment, and this may affect survival analyses [21]. In a pragmatic trial such as the SAFETY trial, it is expected that there will be protocol deviations. However, we have taken measures to limit the deviations through frequent communications with the clinical site personnel, and allowing different methods of follow-up approved by ethics committees to ensure participants attend study visits within the timeframe.

### 4.3. Aggregate Clinical Outcomes and Implications for the Definitive Trial

The mortality rate in the two years of follow-up for this SAFETY trial population (15%) was less than what was expected at the time of study design [11]. Therefore, our initial plan to evaluate overall survival with a non-inferiority design was no longer feasible, given the lower-than-expected death rate and the subsequent requirement for a significantly larger and impractical sample size. The SAE rate, however, was larger than expected at 30%. Finally, the low rates of missed PROMs data at each time point have indicated that the focus for the trial outcomes could be revised as such for the definitive study. With the approval of the SAFETY trial Steering Committee in May of 2024, the SAFETY trial outcome was revised to a hierarchal composite outcome including, in order of importance, mortality, SAEs and PROMIS^®^ Cancer-Anxiety, with the data from this study informing the sample size calculation. The final definitive sample size is 310 participants, which accounts for a conservative 10% dropout for reasons other than death.

### 4.4. Strengths and Limitations

The SAFETY trial was rigorously designed and implemented. Although patient and caregiver blinding cannot be achieved, the objective nature of the study outcomes, such as death and SAEs, minimizes the risk for outcomes bias. In addition, the database is queried regularly, and clinical sites are contacted at regular intervals to provide assistance in ensuring that their data is complete and query-free. This has resulted in a high-quality prospective database for this study and the ongoing definitive phase of the study. The pragmatic nature of the study has allowed surgeons to reassure their patients that they may request additional clinic visits and/or imaging if they feel anxious about their health and this reassurance has maximized study acceptance. Finally, the SAFETY trial clinical sites are widely international, providing generalizability across a variety of healthcare systems and socioeconomic environments.

Despite these strengths, attempts to fund the definitive phase of the study have been exhausted, with multiple unsuccessful submissions to Canadian and American funding agencies. As such, the SAFETY Steering Committee decided on 13 November 2023, that the study would remain unfunded and, therefore, only clinical sites willing to continue enrolment without per-patient payments could continue with enrolment, although follow-up of all previously enrolled patients would continue as per protocol. In addition, the minimum length of follow-up has been reduced by vote of the Steering Committee from five years to three years, to ensure completion of the study in a reasonable time frame. This revision to the study protocol will decrease the amount of information that would otherwise be achieved if all 310 patients completed a full five years of follow-up. However, the median follow-up will likely be 4 years and the outcomes identified by our patient partners as important are now incorporated into the primary outcome. Therefore, the value of the study remains undiminished, and potentially improved by these protocol adjustments.

## 5. Conclusions

The SAFETY trial pilot phase met the progression criteria to move forward to the definitive phase of the trial and the pilot population has been rolled into the definitive phase. Lessons learned include the less-than-expected acceptance rate by eligible patients, and the need to emphasize vigilance in protocol adherence over the two years of the surveillance allocation. The baseline aggregate outcomes have informed a revised primary outcome and sample size calculation. Enrolment for the SAFETY trial (310 participants) was completed in October of 2024 and final follow-up is expected to be completed at the end of 2027. The data ultimately provided by the SAFETY trial will be critical in informing pulmonary surveillance guidelines for STS patients worldwide.

## Figures and Tables

**Table 1:** Feasibility outcomes and progression criteria.

Feasibility Outcome	Progression Criteria
Participant enrolment	Participant retention of at least 85%
Participants’ and clinical sites’ adherence to protocol	Protocol deviations below 15%
Participant follow-up and data quality	Completed follow-up with accurate data collection of at least 85% of study visits

**Table 2:** SAFETY trial conduct procedure.

Step	Visit				Relevant Case Report Forms/Study Materials
Surgery	Pre-Baseline Visit				None
Identification of Patients	Baseline Visit				Screening Log Informed Consent
Assessment of Patient Eligibility					Screening Form Baseline Forms PROMIS Cancer-Anxiety, PROMIS Satisfaction with Social Roles and Activities, and EQ-5D Questionnaires
Randomization to Imaging Modality Frequency (chest CT scan or CXR)					Randomization Form Centralized, 24 h Web-Based Randomization System
Randomization to Surveillance Frequency (clinic visit every 3 months or every 6 months)					Randomization Form Centralized, 24 h Web-Based Randomization System
Study Intervention					
Assessment of Study Intervention and Outcomes	**Treatment Group #1: CXR every 3 months for 2 years**	**Treatment Group #2: CXR every 6 months for 2 years**	**Treatment Group #3: CT every 3 months for 2 years**	**Treatment Group #1: CT every 6 months for 2 years**	
	3M CXR + Study Visit	No Imaging or Study Visit	3M CT + Study Visit	No Imaging or Study Visit	Follow-up Forms
	6M CXR + Study Visit	6M CXR + Study Visit	6M CT + Study Visit	6M CT + Study Visit	Follow-up Forms PROMIS Cancer-Anxiety, PROMIS Satisfaction with Social Roles and Activities, and EQ-5D Questionnaires
	9M CXR + Study Visit	No Imaging or Study Visit	9M CT + Study Visit	No Imaging or Study Visit	Follow-up Forms
	12M CXR + Study Visit	12M CXR + Study Visit	12M CT + Study Visit	12M CT + Study Visit	Follow-up Forms PROMIS Cancer-Anxiety, PROMIS Satisfaction with Social Roles and Activities, and EQ-5D Questionnaires
	15M CXR + Study Visit	No Imaging or Study Visit	15M CT + Study Visit	No Imaging or Study Visit	Follow-up Forms
	18M CXR + Study Visit	18M CXR + Study Visit	18M CT + Study Visit	18M CT + Study Visit	Follow-up Forms PROMIS Cancer-Anxiety, PROMIS Satisfaction with Social Roles and Activities, and EQ-5D Questionnaires
	21M CXR + Study Visit	No Imaging or Study Visit	21M CT + Study Visit	No Imaging or Study Visit	Follow-up Forms
	24M CXR + Study Visit	24M CXR + Study Visit	24M CT + Study Visit	24M CT + Study Visit	Follow-up Forms PROMIS Cancer-Anxiety, PROMIS Satisfaction with Social Roles and Activities, and EQ-5D Questionnaires

M: Month, CT: Commuted Tomography scan, CXR: Chest x-ray, PROMIS: Patient-Reported Outcomes Measurement Information System, EQ-5D: EuroQol Group-5 Dimensions.

**Table 3:** Participating clinical sites in the pilot study.

Hospital	Institution	Country	Date Opened to Enrolment	Pilot Enrolment	Enrolling in Definitive Study
Juravinski Hospital and Cancer Centre	McMaster University	Canada	November 2019	Yes	Yes
Albany Medical Center	Albany College	United States	June 2020	Yes	Yes
UC Davis Medical Center	University of California Davis	United States	June 2020	Yes	Yes
Holden Comprehensive Cancer Center	University of Iowa	United States	September 2020	Yes	Yes
Medical University Graz	Medical University Graz	Austria	October 2020	Yes	Yes
NYU Langone Health Perlmutter Cancer Center	New York University	United States	October 2020	Yes	No
Hôpital Maisonneuve-Rosemont	University of Montreal	Canada	October 2020	Yes	Yes
UF Health Shands Hospital	University of Florida	United States	October 2020	Yes	No
McGill University Health Centre	McGill University	Canada	November 2020	Yes	Yes
Montefiore Medical Center		United States	January 2021	Yes	Yes
L’Hotel-Dieu de Quebec	Laval University	Canada	January 2021	Yes	Yes
Hospital de Clinicas de Porto Alegre		Brazil	March 2021	Yes	Yes
Centro Traumatologico Ortopedico Hospital		Italy	April 2021	Yes	Yes
The Cleveland Clinic and Hillcrest Hospital	Cleveland University	United States	May 2021	Yes	Yes
Huntsman Cancer Institute	University of Utah	United States	June 2021	Yes	Yes
Leiden University Medical Centre	Leiden University	The Netherlands	September 2021	Yes	Yes
Oregon Health and Science University Hospital		United States	September 2021	No	Yes
Hospital Universitario Austral		Argentina	October 2021	No	Yes
Nova Scotia Health	Dalhousie University	Canada	November 2021	Yes	Yes
The Ottawa Hospital	University of Ottawa	Canada	December 2021	No	Yes

**Table 4:** Patient baseline characteristics.

Characteristic			Patients (*n* = 100)
**Gender**			
	Men		52
	Women		48
**Mean age [in years] (SD)**			59.8 (15)
**Type of tumor**			
	Undifferentiated Pleiomorphic Sarcoma		26
	Myxofibrosarcoma		20
	Biphasic Synovial Sarcoma		7
	Undifferentiated Spindle Cell Sarcoma		7
	Pleiomorphic Liposarcoma		6
	Dedifferentiated Liposarcoma		6
	Leiomyosarcoma (non-cutaneous)		5
	Fibroblastic Sarcoma		4
	Spindle Cell Synovial Sarcoma		3
	Undifferentiated Sarcoma		3
	Other		13
**Most common tumor locations**			
	Thigh		33
	Shoulder		12
	Hip		10
	Groin		9
	Knee		8
**Other cancer treatment modalities (in addition to surgery)**			
	No		1
	Yes		99
		Neoadjuvant chemotherapy	6
		Neoadjuvant radiation	77
		Adjuvant chemotherapy	3
		Adjuvant radiation	9

**Table 5:** Protocol deviations in 55 patients. Data are presented as absolute numbers (%).

Protocol Deviation	Patients (*n* = 55)
Potentially ineligible patient enrolled	2 (4)
Unscheduled clinic visit	33 (60)
Missed thoracic imaging	20 (36)
Missed surveillance visit	15 (27)
Unscheduled thoracic imaging	11 (20)
Improper thoracic imaging modality	3 (5)

**Table 6:** Protocol Adherence and Data Completeness.

	Eligible for Follow-Up *	Completed CRFs *n* (%)	Partially Completed CRFs *n* (%)	Outstanding *n* (%)	Missed Visit *n* (%)	Missed PROMs *n* (%)
6 months	89	84 (94)	5 (6)	0 (0)	2 (2)	7 (9)
12 months	84	81 (96)	2 (2)	1 (1)	3 (4)	5 (6)
18 months	81	69 (85)	8 (10)	4 (5)	7 (9)	6 (7)
24 months	80	68 (85)	2 (3)	10 (13)	7 (9)	9 (11)
30 months	73	53 (73)	4 (5)	4 (5)	1 (1)	N/A
36 months	33	21 (64)	2 (6)	4 (12)	2 (6)	4 (12)
42 months	13	9 (69)	0 (0)	1 (8)	0 (0)	N/A
48 months	8	7 (88)	0 (0)	0 (0)	0 (0)	0 (0)
54 months	0	-	-	-	-	N/A
60 months	0	-	-	-	-	N/A

* Number of participants who reached each time point and had not died or withdrawn, as of June 2024. N/A = Not Applicable.

## Data Availability

Data is not available for the pilot phase.

## Supplementary Materials

The following supporting information can be downloaded at: https://www.mdpi.com/article/10.3390/curroncol32120686/s1, Table S1: List of 52 serious adverse events in 30 SAFETY patients.

## Abbreviations

The following abbreviations are used in this manuscript:

STS	Soft-tissue sarcoma
CXR	Chest x-ray
CT	Computed tomography
SAFETY	Surveillance AFter Extremity Tumor SurgerY
RCT	Randomized controlled trial
HiREB	Hamilton integrated Research Ethics Board
CRF	Case report form
SAE	Serious adverse event
PROMIS	Patient-Reported Outcomes Measurement Information System
EQ-5D	EuroQol Group-5 Dimensions
DSMB	Data and safety monitoring board
SD	Standard deviation
PROMs	Patient-Reported Outcome Measures

## Appendix A

*The SAFETY Investigators:

The following persons participated in the SAFETY study:

**Study Principal Investigator:** Michelle Ghert

**Methods Center Staff (McMaster University|Hamilton, Ontario, Canada):** Michelle Ghert, Nathan Evaniew, Paula McKay, Patricia Schneider, Victoria Giglio, Dana Ghanem, Callum MacLeay, Tess Hudson, Hadia Farrukh, Abbey Kunzli, Sheila Sprague, Diane Heels-Ansdell, and Lisa Buckingham

**Steering Committee:** Michelle Ghert (Chair), Mohit Bhandari, Robert Grimer, Gordon Guyatt, James Hayden, Benjamin Miller, Naveen Parasu Nagarajan, Ajay Puri, R. Lor Randall, Lehana Thabane, Robert Turcotte, Michiel van de Sande, Roberto Vélez, David Wilson, Kevin Zbuk, Sheila Sprague, Peter Fantozzi, Jean-Eric Tarride, Peter Ferguson, and Georges Basile

**Data Safety Monitoring Board:** Peter Rose (Chair), Brian Brigman, Stephanie Pritchett, Cynthia Rankins, Brittany Siontis, and Kevin Thorpe

**Clinical Sites:**

*Pilot Phase and Definitive Phase Sites with Patient Enrolment*

**Juravinski Hospital and Cancer Centre (Hamilton, Ontario)**: Michelle Ghert, David Wilson, Scott Evans, Kimmen Quan, Valerie Francescutti, Mira Goldberg, Michele Bertothy, Christopher Coroneos, Samantha Sigurdson, Julianna Sienna, Pierluca Zecchetto, Victoria Giglio, Dana Ghanem, Callum MacLeay, Tess Hudson, Hadia Farrukh, and Abbey Kunzli

**McGill University Health Centre (Montreal, Quebec)**: Robert Turcotte, Ahmed Aoude, Anthony Bozzo, Mireille Dessureault, and Nadine Zablith

**Hôpital Maisonneuve-Rosemont (Montreal, Quebec)**: Georges Basile, Marc Isler, Sophie Mottard, Janie Barry, and Hugo Saint-Yves

**CHU de Québec—Université Laval (Québec, Quebec)**: Annie Arteau, Norbert Dion, Sylvie Turmel, and Laura Zuviri

**Huntsman Cancer Institute (Salt Lake City, Utah)**: Kevin Jones, John Groundland, Jacqueline Hart, and Cameron Skiby

**Hospital Universitario Austral (Buenos Aires, Argentina)**: Marcos Galli Serra, Walter Parizzia, Bruno Terrarosa, Manuel de Elias, Manglio Miguel Rizzo, Mercedes Gunning, Veronica Romero Brancato, and María Santillan

**Holden Comprehensive Cancer Center (Iowa City, Iowa)**: Benjamin Miller, Jill Kain, Tammy Smith, and Abigail Grothe

**Montefiore Medical Center (Bronx, New York)**: David Geller, Bang Hoang, Rui Yang, Janet Tingling, Dana Kamens, Robert Schneider, and Sung-Suk Chae

**Albany Medical Center (Albany, New York)**: Matthew DiCaprio, Humza Murtaza, and Bradford Palmer

**Oregon Health and Science University Hospital (Portland, Oregon)**: Yee-Cheen Doung, James Hayden, Kenneth Gundle, Duncan Ramsey, Christopher Ryan, Lara Davis, Rebecca Smith, and Steven Shelofsky

**University of Florida Health Shands Hospital (Gainesville, Florida)**: Andre Spiguel, Mark Scarborough, Charles Parker Gibbs, Johanna Carmona, and Aimee Struk

**Leiden University Medical Center (Leiden, Netherlands)**: Michiel van de Sande, Robert van der Wal, Demien Broekhuis, Jos van der Hage, Desirée Dorleijn, Marloes van Duijvenbode, and V. Bahadoer

**The Cleveland Clinic (Cleveland, Ohio)**: Nathan Mesko, Lukas Nystrom, Zachary Burke, Kristi Mareth, Rachael Laubacher, Andre Roman, Michael Johnson, Matthew Rerko, Kierstyn Hayden, Heather Keaney, Maria Clark, Skylar Evans, Mingo Rolince, and Erin Sites

**Hartford Healthcare (Hartford, Connecticut):** Adam Lindsay, Emily Shearier, Oisharya Dasgupta, Bethany Samperi, and Isabel Lubin

**Hospital de Clínicas de Porto Alegre (HCPA) (Porto Alegre, Brazil):** Ricardo Gehrke Becker, Bruno Antunes Pereira, and Julie Francine Cerutti Santos Pestilho

**LKH—Universitätsklinikum Graz (Graz, Austria)**: Marko Bergovec, Andreas Leithner, Dimosthenis Andreou, Robert Piber, Marina Bergovec, Andrea Fink, Pablo Zardoya-Laguardia, Arlinda Lushtaku, Sandra Karre, Catharina Friedl, and Julia Geyer

**UC Davis Medical Center (Davis, California):** Steven Thorpe, R. Lor Randall, Julia Martin, Abigail Inkster, Chancey Sweeney, Gaby Zumaran, and Maya Porter

**NYU Langone Health Perlmutter Cancer Center (New York, New York):** Karim Masrouha, Debra Sala, Joie Cooper, and Micaela Tomaro

**AOU Città della Salute e della Scienza di Torino, CTO Hospital (Torino, Italy)**: Michele Boffano, Raimondo Piana, Ugo Albertini, Elena Boux, Andrea Ferro, Stefano Marone, Pietro Pellegrino, Nicola Ratto, and Simone De Meo

**Nova Scotia Health (Halifax, Nova Scotia):** David Wilson, Michael Biddulph, Kelly Trask, and Curtis Giacomantonio

*Sites Enrolling in the Definitive Trial Only*

**Mount Sinai Hospital (Toronto, Ontario)**: Peter Ferguson, Jay Wunder, Kim Tsoi, Anthony Griffin, and Raghda AlAtia

**The Ottawa Hospital (Ottawa, Ontario)**: Joel Werier, Hesham Abdelbary, and Yusra Kassim

**Hospital Universitario Vall d’Hebron (Barcelona, Spain)**: Roberto Velez, María Garcia, and Angela Roig Molina

**Dartmouth-Hitchcock Medical Center (Lebanon, New Hamphire):** Eric Henderson, Andrew Loehrer, Casey O’Quinn, Polly Folsom, Ashley Burns, Grace Lachance, Carmeleta Beidler, Tayyaba Sarwar, Sarah Dilanni, and Jacob Dubien

**Parkview Cancer Institute (Fort Wayne, Indiana):** Christopher Johnson, Emily Myers, Zhentao Zhang, Lydia Harman, Karen Kreiger, Caylin Boles, Jon Lehrman, Ashley Romine, Marissa White, Tarryn Degroff, Lev Inger, Pamela Meyer, Angela Hamman, Jilian Allis, Kelsey Townsend, Kari Schultz, and Breanna Henry

**St. Vincent’s Hospital Melbourne (Melbourne, Australia):** Peter Choong and Angela Cochrane

**University Malaya Kuala Lumpur (Kuala Lumpur, Malaysia):** Vivek Ajit Singh, Nor Faissal Yasin, and Priscilla Josephine Samuel

**Texas Tech Health Sciences Center (El Paso, Texas):** Rajiv Rajani, Brianna Godinez, Diego Sanchez, Nikit Venishetty, Lissete Sanchez, Daniel De Leon, Brenda Castillo, and Janeth Sandoval

**Cliniques Universitaires Saint Luc (Brussels, Belgium):** Thomas Schubert, Filomena Mazzeo, Xavier Geets, Zeyneb El Mouch, Nathalie Blondeel, and Kim Henquin

**Allegheny Health Network Research Institute (Pittsburgh, Pennsylvania):** Lisa Ercolano, Alethea Carr, and Myles Forsyth

**Karolinska University Hospital (Stockholm, Sweden):** Panagiotis Tsagkozis, and Susanne Olsson

**University of Chicago Medicine and Biological Sciences Division (Chicago, Illinois):** Tessa Balach, Rex Haydon, Olivia Keaveny, Carla Tisdale, and Michael Koch

**UWashington Fred Hutchinson Cancer Center (Seattle, Washington):** Elyse Brinkmann, Paige Ourada, Elizabeth Loggers, Jennifer Hamilton, Steven Ginsberg, Elizabeth Sieber, Amy Morgan, Stephanie Doyle, Niall Morin, David Siu, Vina Nguyen, Rylee Johnson, Robyn Kunsman, Eric Damiana, Shannon Maxwell, and Lauren Smith

**Helios Klinikum Berlin (Berlin, Germany):** Maya Niethard, Per-Ulf Tunn, Janina Kuhnke

**Centro Hospitalar e Universitario de Coimbra (Coimbra, Portugal):** Joao Paulo Fonseca de Freitas, Catarina Neves
