# Supplementary material for: The Surveillance After Extremity Tumor Surgery (SAFETY) Pilot International Multi-Center Randomized Controlled Trial
Source: Curr Oncol. 2025 Dec 4;32(12):686. doi: 10.3390/curroncol32120686 (PMC12731459; doi:10.3390/curroncol32120686)
Supplement: Supplementary file 1 [file curroncol-32-00686-s001.zip › curroncol-3932213-supplementary.pdf]

Table S1: List of 52 serious adverse events in 30 SAFETY patients

Serious Adverse Event Description	Location (if applicable)
Sprain	Left knee
Foul-smelling growth	Below axilla posteriorly (10x4 cm) in middle of previous excision
Pain	Right upper quadrant
Worsening osteoarthritis	Left hip
Nodule	Wound site (left thigh)
Malignant effusion	Lungs (bilateral)
Hydropneumothorax	Right lung
Anemia	
Anorexia-cachexia syndrome due to advanced cancer	
Anemia	
Meniscal tears	Left knee
Acute cholecystitis	
Tendonitis, tendon tear	Left shoulder, bicep
Cellulitis	Surgical site (right thigh)
Heart blockage – triple vessel coronary calcifications	
Melanoma	Right lateral wrist
Complications from Parkinson's disease	
Decreased urine output	
Shortness of breath	
Gallstones	
Seroma (10 cm long, 4 cm high)	Tumor site (axilla)
Compression of thoracic spinal cord	T6
Leukocytosis, hyponatremia	
Edema post biopsy	Right hip
Surgical site infection post-op for local recurrence	Drain site (right groin)
Surgical site infection	Left ankle
Cellulitis	Left gluteal region (site of cyst that was biopsied)
Pain	Tumor site (left leg – ankle) and buttock
Acute on chronic perforated appendicitis	
Worsening polyp tubulovillous adenoma – benign	Ascending colon polyp measuring 12 x 17 mm
Post-op bowel obstruction	Small bowel/intestine
Mural thrombus	Infrarenal aorta
Medication reaction from Fragmin (Severe headache, severe shortness of breath, burning in feet)	
Fall and pathological fractures	Bilateral femur, left humerus
Massive cerebrovascular accident	
Broken surgical plate	Femoral
Pathologic fracture	Femur
Hernia	Flap Site (Flank)

Hemorrhage	Liver
Pain	Lower back
Nausea	
Pain	Abdominal
Fever cause by covid-19	
Cellulitis	Right posterior thigh
"Balloon procedure lower extremity for ischemia"	Surgical extremity
Seroma of musculoskeletal structure after musculoskeletal system procedure	Left thigh
Squamous cell carcinoma	Left ear
Covid-19 infection	
Patella fracture	Right side
Femur head fracture	Femur head – right leg
Abscess	Pectoral
Atypical lipomatous tumour	Right thigh
